# Supplementary material for: Shifting temporal trends and disparities in sarcoidosis mortality in the United States: A retrospective analysis from 1999 to 2020
Source: PLoS One. 2025 Jan 10;20(1):e0317237. doi: 10.1371/journal.pone.0317237 (PMC11723600; doi:10.1371/journal.pone.0317237)
Supplement: S1 Table — (DOCX) [file pone.0317237.s001.docx]

**S1 Table –Sarcoidosis related Deaths, Stratified by Sex, in the United States, 1999 to 2020**

| **Year** | **Overall Deaths** | **Male Deaths** | **Female Deaths** | **Population** |
| --- | --- | --- | --- | --- |
| 1999 | 1072 | 415 | 657 | 279,040,168 |
| 2000 | 1299 | 476 | 823 | 281,421,906 |
| 2001 | 1377 | 467 | 910 | 284,968,955 |
| 2002 | 1473 | 549 | 924 | 287,625,193 |
| 2003 | 1475 | 546 | 929 | 290,107,933 |
| 2004 | 1432 | 552 | 880 | 292,805,298 |
| 2005 | 1519 | 575 | 944 | 295,516,599 |
| 2006 | 1569 | 570 | 999 | 298,379,912 |
| 2007 | 1578 | 588 | 990 | 301,231,207 |
| 2008 | 1566 | 618 | 948 | 304,093,966 |
| 2009 | 1688 | 670 | 1018 | 306,771,529 |
| 2010 | 1692 | 683 | 1009 | 308,745,538 |
| 2011 | 1779 | 713 | 1066 | 311,591,917 |
| 2012 | 1768 | 722 | 1046 | 313,914,040 |
| 2013 | 1822 | 766 | 1056 | 316,128,839 |
| 2014 | 1878 | 776 | 1102 | 318,857,056 |
| 2015 | 1940 | 814 | 1126 | 321,418,820 |
| 2016 | 1996 | 819 | 1177 | 323,127,513 |
| 2017 | 2085 | 908 | 1177 | 325,719,178 |
| 2018 | 2140 | 958 | 1182 | 327,167,434 |
| 2019 | 2185 | 913 | 1272 | 328,239,523 |
| 2020 | 2623 | 1149 | 1474 | 329,484,123 |
| Total | 37956 | 15247 | 22709 | 6746356647 |
