# Supplementary material for: Shifting temporal trends and disparities in sarcoidosis mortality in the United States: A retrospective analysis from 1999 to 2020
Source: PLoS One. 2025 Jan 10;20(1):e0317237. doi: 10.1371/journal.pone.0317237 (PMC11723600; doi:10.1371/journal.pone.0317237)
Supplement: S2 Table — (DOCX) [file pone.0317237.s002.docx]

**S2 Table – Sarcoidosis related Mortality, Stratified by Place of Death in the United States, 1999 to 2020**

| **Year** | **Medical Facility Deaths** | **Home Deaths** | **Hospice Deaths** | **Nursing Home Deaths** |
| --- | --- | --- | --- | --- |
| 1999 | 785 | 180 | Missing | 72 |
| 2000 | 976 | 222 | Missing | 73 |
| 2001 | 1006 | 255 | Missing | 87 |
| 2002 | 1053 | 269 | Missing | 100 |
| 2003 | 1058 | 261 | Suppressed | 102 |
| 2004 | 1003 | 271 | Suppressed | 99 |
| 2005 | 1031 | 304 | 15 | 112 |
| 2006 | 1046 | 339 | 17 | 111 |
| 2007 | 1044 | 345 | 32 | 101 |
| 2008 | 997 | 340 | 34 | 111 |
| 2009 | 1060 | 372 | 30 | 112 |
| 2010 | 1040 | 397 | 53 | 132 |
| 2011 | 1099 | 426 | 66 | 115 |
| 2012 | 1052 | 441 | 73 | 121 |
| 2013 | 1080 | 432 | 88 | 142 |
| 2014 | 1084 | 500 | 77 | 158 |
| 2015 | 1071 | 530 | 131 | 149 |
| 2016 | 1133 | 517 | 120 | 175 |
| 2017 | 1148 | 554 | 143 | 175 |
| 2018 | 1115 | 660 | 134 | 160 |
| 2019 | 1113 | 668 | 153 | 187 |
| 2020 | 1371 | 837 | 126 | 195 |
| Total | 23365 | 9120 | 1295 | 2789 |
