# Supplementary material for: Shifting temporal trends and disparities in sarcoidosis mortality in the United States: A retrospective analysis from 1999 to 2020
Source: PLoS One. 2025 Jan 10;20(1):e0317237. doi: 10.1371/journal.pone.0317237 (PMC11723600; doi:10.1371/journal.pone.0317237)
Supplement: S3 Table — (DOCX) [file pone.0317237.s003.docx]

**S3 Table: Overall and Sex‐Stratified Sarcoidosis related Age-Adjusted Mortality Rates per 1,000,000 in the United States, 1999 to 2020**

| **Year** | **Overall Age-Adjusted Rate (Lower CI - Upper CI)** | **Female Age-Adjusted Rate (Lower CI - Upper CI)** | **Male Age-Adjusted Rate (Lower CI - Upper CI)** |
| --- | --- | --- | --- |
| 1999 | 3.9 (3.7 - 4.1) | 4.4 (4.1 - 4.8) | 3.2 (2.9 - 3.6) |
| 2000 | 4.7 (4.4 - 4.9) | 5.5 (5.1 - 5.9) | 3.6 (3.3 - 4.0) |
| 2001 | 4.8 (4.6 - 5.1) | 6.0 (5.6 - 6.4) | 3.5 (3.2 - 3.9) |
| 2002 | 5.1 (4.8 - 5.3) | 5.9 (5.6 - 6.3) | 4.0 (3.7 - 4.4) |
| 2003 | 5.0 (4.8 - 5.3) | 5.9 (5.5 - 6.2) | 4.0 (3.6 - 4.3) |
| 2004 | 4.8 (4.5 - 5.0) | 5.5 (5.1 - 5.8) | 3.9 (3.6 - 4.3) |
| 2005 | 5.0 (4.7 - 5.2) | 5.7 (5.4 - 6.1) | 4.0 (3.7 - 4.4) |
| 2006 | 5.0 (4.8 - 5.3) | 6.0 (5.6 - 6.4) | 3.9 (3.6 - 4.3) |
| 2007 | 5.0 (4.7 - 5.2) | 5.8 (5.4 - 6.1) | 4.0 (3.7 - 4.4) |
| 2008 | 4.8 (4.6 - 5.1) | 5.4 (5.1 - 5.8) | 4.1 (3.8 - 4.4) |
| 2009 | 5.1 (4.9 - 5.4) | 5.7 (5.4 - 6.1) | 4.4 (4.0 - 4.7) |
| 2010 | 5.0 (4.8 - 5.3) | 5.6 (5.2 - 5.9) | 4.4 (4.1 - 4.7) |
| 2011 | 5.3 (5.0 - 5.5) | 5.8 (5.5 - 6.2) | 4.6 (4.2 - 4.9) |
| 2012 | 5.1 (4.9 - 5.3) | 5.5 (5.2 - 5.9) | 4.5 (4.1 - 4.8) |
| 2013 | 5.1 (4.9 - 5.4) | 5.4 (5.1 - 5.8) | 4.7 (4.3 - 5.0) |
| 2014 | 5.2 (5.0 - 5.4) | 5.6 (5.2 - 5.9) | 4.6 (4.3 - 5.0) |
| 2015 | 5.3 (5.1 - 5.6) | 5.7 (5.3 - 6.0) | 4.8 (4.5 - 5.1) |
| 2016 | 5.3 (5.0 - 5.5) | 5.7 (5.4 - 6.0) | 4.7 (4.4 - 5.1) |
| 2017 | 5.4 (5.2 - 5.7) | 5.6 (5.3 - 6.0) | 5.1 (4.7 - 5.4) |
| 2018 | 5.5 (5.2 - 5.7) | 5.4 (5.1 - 5.7) | 5.4 (5.0 - 5.7) |
| 2019 | 5.5 (5.2 - 5.7) | 5.8 (5.5 - 6.2) | 5.0 (4.7 - 5.3) |
| 2020 | 6.4 (6.2 - 6.7) | 6.6 (6.2 - 6.9) | 6.1 (5.7 - 6.5) |
|  |  |  |  |
