# Supplementary material for: Shifting temporal trends and disparities in sarcoidosis mortality in the United States: A retrospective analysis from 1999 to 2020
Source: PLoS One. 2025 Jan 10;20(1):e0317237. doi: 10.1371/journal.pone.0317237 (PMC11723600; doi:10.1371/journal.pone.0317237)
Supplement: S4 Table — (DOCX) [file pone.0317237.s004.docx]

**S4 Table : Annual percent change (APC) of Sarcoidosis related Age-Adjusted Mortality Rates per 1,000,000 in the United States, 1999 to 2020, 1999 to 2020**

| Year Interval | APC (95% CI) |
| --- | --- |
| Overall |  |
| 1999-2001 | 9.66 (2.26-15.92) |
| 2001-2018 | 0.52 (-0.65-0.77) |
| 2018-2020 | 8.54 (2.66-11.52) |
| Men |  |
| 1999-2018 | 2.01 (-0.93-7.27) |
| 2018-2020 | 7.43 (2.05-11.08) |
| Women |  |
| 1999-2001 | 13.43 (5.85-20.95) |
| 2001-2018 | -0.45 (-1.1- -0.16) |
| 2018-2020 | 9.20 (1.95-12.54) |
| Northeast |  |
| 1999-2018 | 0.43 (-1.18-0.89) |
| 2018-2020 | 9.04 (1.15-15.78) |
| Midwest |  |
| 1999-2020 | 1.54 (0.98-2.17) |
| South |  |
| 1999-2001 | 12.37 (4.07-19.14) |
| 2001-2018 | 0.12 (-0.57-0.42) |
| 2018-2020 | 9.64 (3.06-13.20) |
| West |  |
| 1999-2003 | 11.67 (6.35-25.38) |
| 2003-2010 | -1.32 (-7.21-0.62) |
| 2010-2020 | 2.78 (1.75-7.32) |
| Nonmetropolitan areas |  |
| 1999-2018 | 1.34 (-0.65-2.21) |
| 2018-2020 | 15.29 (2.09-21.89) |
| Metropolitan area |  |
| 1999-2001 | 9.66 (1.13-18.65) |
| 2001-2018 | 0.39 (-3.93-0.66) |
| 2018-2020 | 7.09 (1.05-10.55) |
| Black or African American |  |
| 1999-2001 | 9.46 (-0.20-20.52) |
| 2001-2018 | -0.95 (-6.70 - -0.58) |
| 2018-2020 | 10.62 (0.82-15.88) |
| White |  |
| 1999-2001 | 12.13 (3.27-19.81) |
| 2001-2018 | 2.19 (-0.42-2.45) |
| 2018-2020 | 6.57 (2.65-9.16) |
| Hispanic or Latino |  |
| 1999-2020 | 0.99 (-0.12-2.64) |

APC = annual percent change
