# Supplementary material for: Shifting temporal trends and disparities in sarcoidosis mortality in the United States: A retrospective analysis from 1999 to 2020
Source: PLoS One. 2025 Jan 10;20(1):e0317237. doi: 10.1371/journal.pone.0317237 (PMC11723600; doi:10.1371/journal.pone.0317237)
Supplement: S5 Table — (DOCX) [file pone.0317237.s005.docx]

**S5 Table: State‐Stratified Sarcoidosis related Age-Adjusted Mortality Rates per 1,000,000 in the United States, 1999 to 2020**

| **State** | **Age Adjusted Rate (Lower CI - Upper CI)** |
| --- | --- |
| Alabama | 8.2 (7.6 - 8.7) |
| Alaska | 6 (4.7 - 7.6) |
| Arizona | 2.1 (1.8 - 2.3) |
| Arkansas | 4.6 (4.1 - 5.2) |
| California | 3.2 (3.1 - 3.3) |
| Colorado | 4.2 (3.8 - 4.6) |
| Connecticut | 4.9 (4.4 - 5.3) |
| Delaware | 7.8 (6.6 - 9) |
| District of Columbia | 21.9 (19.3 - 24.4) |
| Florida | 3.9 (3.8 - 4.1) |
| Georgia | 7.1 (6.7 - 7.4) |
| Hawaii | 0.7 (0.4 - 1) |
| Idaho | 3.5 (2.9 - 4.2) |
| Illinois | 5.1 (4.8 - 5.3) |
| Indiana | 4.6 (4.3 - 5) |
| Iowa | 3.7 (3.3 - 4.2) |
| Kansas | 4.3 (3.8 - 4.8) |
| Kentucky | 3.9 (3.5 - 4.2) |
| Louisiana | 6.3 (5.8 - 6.7) |
| Maine | 5 (4.3 - 5.8) |
| Maryland | 10.7 (10.2 - 11.3) |
| Massachusetts | 3.9 (3.6 - 4.2) |
| Michigan | 6.9 (6.6 - 7.3) |
| Minnesota | 4.5 (4.2 - 4.9) |
| Mississippi | 8.4 (7.7 - 9.1) |
| Missouri | 4.5 (4.1 - 4.8) |
| Montana | 3.7 (2.9 - 4.5) |
| Nebraska | 3.9 (3.3 - 4.5) |
| Nevada | 2.8 (2.4 - 3.3) |
| New Hampshire | 3.8 (3.1 - 4.4) |
| New Jersey | 6 (5.7 - 6.3) |
| New Mexico | 1.7 (1.4 - 2.1) |
| New York | 6 (5.7 - 6.2) |
| North Carolina | 9 (8.6 - 9.4) |
| North Dakota | 3.2 (2.4 - 4.2) |
| Ohio | 6.3 (6 - 6.6) |
| Oklahoma | 4 (3.6 - 4.4) |
| Oregon | 3.9 (3.5 - 4.3) |
| Pennsylvania | 5.5 (5.3 - 5.8) |
| Rhode Island | 4.9 (4 - 5.7) |
| South Carolina | 10.7 (10.1 - 11.3) |
| South Dakota | 3.5 (2.8 - 4.4) |
| Tennessee | 5.8 (5.4 - 6.2) |
| Texas | 3.9 (3.7 - 4.1) |
| Utah | 4.8 (4.1 - 5.4) |
| Vermont | 6.5 (5.2 - 7.7) |
| Virginia | 7.4 (7 - 7.8) |
| Washington | 5 (4.7 - 5.4) |
| West Virginia | 4.1 (3.5 - 4.6) |
| Wisconsin | 5.4 (5 - 5.7) |
| Wyoming | 3.7 (2.7 - 4.9) |
|  |  |
