# Supplementary material for: Shifting temporal trends and disparities in sarcoidosis mortality in the United States: A retrospective analysis from 1999 to 2020
Source: PLoS One. 2025 Jan 10;20(1):e0317237. doi: 10.1371/journal.pone.0317237 (PMC11723600; doi:10.1371/journal.pone.0317237)
Supplement: S6 Table — (DOCX) [file pone.0317237.s006.docx]

**S6 Table : Census Region‐Stratified Sarcoidosis related Age-Adjusted Mortality Rates per 1,000,000 in the United States, 1999 to 2020**

| Year | Census Region | Age Adjusted Rate (Lower CI - Upper CI) |
| --- | --- | --- |
| 1999 | Northeast | 4.7 (4.1 - 5.3) |
| 2000 | Northeast | 5.2 (4.6 - 5.8) |
| 2001 | Northeast | 5.2 (4.6 - 5.8) |
| 2002 | Northeast | 6.0 (5.3 - 6.6) |
| 2003 | Northeast | 4.9 (4.3 - 5.5) |
| 2004 | Northeast | 5.1 (4.5 - 5.7) |
| 2005 | Northeast | 5.0 (4.4 - 5.5) |
| 2006 | Northeast | 5.3 (4.7 - 5.9) |
| 2007 | Northeast | 5.3 (4.7 - 5.9) |
| 2008 | Northeast | 5.0 (4.5 - 5.6) |
| 2009 | Northeast | 5.4 (4.8 - 6.0) |
| 2010 | Northeast | 5.5 (4.9 - 6.0) |
| 2011 | Northeast | 5.3 (4.8 - 5.9) |
| 2012 | Northeast | 5.6 (5.0 - 6.2) |
| 2013 | Northeast | 5.3 (4.7 - 5.8) |
| 2014 | Northeast | 5.4 (4.8 - 5.9) |
| 2015 | Northeast | 5.9 (5.3 - 6.5) |
| 2016 | Northeast | 5.5 (5.0 - 6.1) |
| 2017 | Northeast | 5.4 (4.8 - 5.9) |
| 2018 | Northeast | 5.7 (5.1 - 6.3) |
| 2019 | Northeast | 5.6 (5.1 - 6.2) |
| 2020 | Northeast | 6.8 (6.2 - 7.4) |
| 1999 | Midwest | 3.9 (3.4 - 4.4) |
| 2000 | Midwest | 4.9 (4.3 - 5.4) |
| 2001 | Midwest | 4.8 (4.2 - 5.3) |
| 2002 | Midwest | 4.9 (4.4 - 5.4) |
| 2003 | Midwest | 4.9 (4.4 - 5.5) |
| 2004 | Midwest | 4.5 (4.0 - 5.0) |
| 2005 | Midwest | 5.2 (4.7 - 5.8) |
| 2006 | Midwest | 5.0 (4.4 - 5.5) |
| 2007 | Midwest | 5.4 (4.9 - 6.0) |
| 2008 | Midwest | 4.9 (4.4 - 5.5) |
| 2009 | Midwest | 4.8 (4.3 - 5.3) |
| 2010 | Midwest | 5.1 (4.6 - 5.7) |
| 2011 | Midwest | 5.2 (4.7 - 5.8) |
| 2012 | Midwest | 5.0 (4.5 - 5.6) |
| 2013 | Midwest | 5.6 (5.1 - 6.2) |
| 2014 | Midwest | 5.4 (4.9 - 5.9) |
| 2015 | Midwest | 5.6 (5.0 - 6.1) |
| 2016 | Midwest | 5.5 (5.0 - 6.0) |
| 2017 | Midwest | 6.0 (5.5 - 6.6) |
| 2018 | Midwest | 5.7 (5.2 - 6.3) |
| 2019 | Midwest | 5.5 (5.0 - 6.0) |
| 2020 | Midwest | 6.8 (6.2 - 7.3) |
| 1999 | South | 4.5 (4.1 - 4.9) |
| 2000 | South | 5.6 (5.1 - 6.0) |
| 2001 | South | 5.8 (5.3 - 6.2) |
| 2002 | South | 6.1 (5.6 - 6.5) |
| 2003 | South | 6.1 (5.7 - 6.6) |
| 2004 | South | 5.6 (5.2 - 6.1) |
| 2005 | South | 5.9 (5.5 - 6.4) |
| 2006 | South | 6.0 (5.5 - 6.4) |
| 2007 | South | 5.6 (5.1 - 6.0) |
| 2008 | South | 5.8 (5.4 - 6.2) |
| 2009 | South | 6.3 (5.8 - 6.7) |
| 2010 | South | 6.1 (5.6 - 6.5) |
| 2011 | South | 6.3 (5.8 - 6.7) |
| 2012 | South | 6.1 (5.6 - 6.5) |
| 2013 | South | 5.9 (5.5 - 6.3) |
| 2014 | South | 6.1 (5.7 - 6.5) |
| 2015 | South | 6.1 (5.7 - 6.5) |
| 2016 | South | 6.0 (5.6 - 6.4) |
| 2017 | South | 6.0 (5.6 - 6.4) |
| 2018 | South | 6.1 (5.7 - 6.6) |
| 2019 | South | 6.4 (6.0 - 6.8) |
| 2020 | South | 7.4 (6.9 - 7.8) |
| 1999 | West | 2.1 (1.7 - 2.5) |
| 2000 | West | 2.4 (2.0 - 2.8) |
| 2001 | West | 3.0 (2.6 - 3.5) |
| 2002 | West | 2.8 (2.4 - 3.3) |
| 2003 | West | 3.4 (2.9 - 3.8) |
| 2004 | West | 3.4 (2.9 - 3.8) |
| 2005 | West | 3.2 (2.8 - 3.6) |
| 2006 | West | 3.4 (2.9 - 3.8) |
| 2007 | West | 3.3 (2.9 - 3.8) |
| 2008 | West | 3.0 (2.5 - 3.4) |
| 2009 | West | 3.2 (2.8 - 3.7) |
| 2010 | West | 3.0 (2.6 - 3.4) |
| 2011 | West | 3.5 (3.1 - 4.0) |
| 2012 | West | 3.2 (2.8 - 3.7) |
| 2013 | West | 3.3 (2.9 - 3.7) |
| 2014 | West | 3.3 (2.9 - 3.7) |
| 2015 | West | 3.3 (2.9 - 3.7) |
| 2016 | West | 3.6 (3.2 - 4.0) |
| 2017 | West | 3.7 (3.3 - 4.1) |
| 2018 | West | 3.8 (3.4 - 4.3) |
| 2019 | West | 4.1 (3.7 - 4.6) |
| 2020 | West | 4.3 (3.9 - 4.8) |
