# Supplementary material for: Shifting temporal trends and disparities in sarcoidosis mortality in the United States: A retrospective analysis from 1999 to 2020
Source: PLoS One. 2025 Jan 10;20(1):e0317237. doi: 10.1371/journal.pone.0317237 (PMC11723600; doi:10.1371/journal.pone.0317237)
Supplement: S7 Table — (DOCX) [file pone.0317237.s007.docx]

**S7 Table: Urbanization‐Stratified Sarcoidosis related Age-Adjusted Mortality Rates per 1,000,000 in the United States, 1999 to 2020**

| Year | Non-Metro Age Adjusted Rate (Lower CI - Upper CI) | Metro Age Adjusted Rate (Lower CI - Upper CI) |
| --- | --- | --- |
| 1999 | 3.4 (2.8 - 3.9) | 4.0 (3.7 - 4.2) |
| 2000 | 3.7 (3.2 - 4.3) | 4.9 (4.6 - 5.1) |
| 2001 | 4.3 (3.7 - 4.9) | 4.9 (4.7 - 5.2) |
| 2002 | 4.1 (3.6 - 4.7) | 5.3 (5.0 - 5.6) |
| 2003 | 4.0 (3.4 - 4.5) | 5.2 (4.9 - 5.5) |
| 2004 | 4.0 (3.4 - 4.5) | 4.9 (4.6 - 5.2) |
| 2005 | 4.8 (4.2 - 5.4) | 5.0 (4.7 - 5.3) |
| 2006 | 4.7 (4.1 - 5.3) | 5.1 (4.8 - 5.4) |
| 2007 | 4.1 (3.5 - 4.6) | 5.2 (4.9 - 5.4) |
| 2008 | 4.2 (3.7 - 4.8) | 5.0 (4.7 - 5.2) |
| 2009 | 4.7 (4.2 - 5.3) | 5.2 (4.9 - 5.5) |
| 2010 | 4.6 (4.0 - 5.2) | 5.2 (4.9 - 5.4) |
| 2011 | 4.9 (4.3 - 5.5) | 5.4 (5.1 - 5.6) |
| 2012 | 4.9 (4.3 - 5.5) | 5.2 (4.9 - 5.4) |
| 2013 | 4.9 (4.3 - 5.5) | 5.2 (4.9 - 5.4) |
| 2014 | 4.9 (4.3 - 5.5) | 5.2 (5.0 - 5.5) |
| 2015 | 4.7 (4.2 - 5.3) | 5.4 (5.1 - 5.7) |
| 2016 | 4.6 (4.1 - 5.2) | 5.4 (5.2 - 5.7) |
| 2017 | 5.1 (4.5 - 5.7) | 5.5 (5.2 - 5.8) |
| 2018 | 5.0 (4.5 - 5.6) | 5.5 (5.3 - 5.8) |
| 2019 | 5.5 (4.8 - 6.1) | 5.5 (5.2 - 5.8) |
| 2020 | 7.0 (6.3 - 7.7) | 6.3 (6.0 - 6.6) |
