# Supplementary material for: Shifting temporal trends and disparities in sarcoidosis mortality in the United States: A retrospective analysis from 1999 to 2020
Source: PLoS One. 2025 Jan 10;20(1):e0317237. doi: 10.1371/journal.pone.0317237 (PMC11723600; doi:10.1371/journal.pone.0317237)
Supplement: S8 Table — (DOCX) [file pone.0317237.s008.docx]

**S8 Table: State‐Stratified Sarcoidosis related Age-Adjusted Mortality Rates per 1,000,000 in the United States, 1999 to 2020**

| Year | NH White | NH Black or African American | NH American Indian or Alaska Native | Hispanic | NH Asian or Pacific Islander |
| --- | --- | --- | --- | --- | --- |
| 1999 | 1.9 (1.7-2.1) | 21.5 (19.8-23.2) | N/A | 1.2 (0.7-1.9) | N/A |
| 2000 | 2.3 (2.1-2.5) | 25 (23.2-26.9) | N/A | 1.9 (1.3-2.8) | N/A |
| 2001 | 2.5 (2.3-2.7) | 25.3 (23.5-27.1) | N/A | 1.4 (0.9-2) | N/A |
| 2002 | 2.6 (2.4-2.8) | 27.4 (25.5-29.3) | N/A | 1.3 (0.8-1.9) | N/A |
| 2003 | 2.4 (2.2-2.6) | 27.5 (25.6-29.3) | N/A | 1.9 (1.4-2.6) | Unreliable (0.4-1.7) |
| 2004 | 2.5 (2.3-2.7) | 24.8 (23-26.6) | N/A | 1.7 (1.2-2.4) | N/A |
| 2005 | 2.7 (2.5-2.9) | 25.2 (23.5-27) | N/A | 1.8 (1.3-2.4) | N/A |
| 2006 | 2.8 (2.6-3) | 25.4 (23.7-27.2) | N/A | 1.5 (1.1-2.1) | N/A |
| 2007 | 2.9 (2.7-3.1) | 24.3 (22.6-26) | N/A | 1.6 (1.1-2.2) | N/A |
| 2008 | 2.8 (2.6-3) | 23.2 (21.6-24.9) | N/A | 1.2 (0.8-1.7) | Unreliable (0.7-2.1) |
| 2009 | 2.9 (2.7-3.1) | 25.3 (23.6-27) | N/A | 1.4 (0.9-1.9) | Unreliable (0.6-2) |
| 2010 | 3 (2.8-3.2) | 23.7 (22.1-25.3) | Unreliable (1.9-7.4) | 1.8 (1.3-2.4) | N/A |
| 2011 | 3.1 (2.9-3.3) | 24.4 (22.8-26.1) | N/A | 1.8 (1.3-2.3) | Unreliable (0.4-1.5) |
| 2012 | 3.2 (3-3.4) | 22.9 (21.3-24.5) | N/A | 1.4 (1-1.9) | Unreliable (0.4-1.4) |
| 2013 | 3.1 (2.9-3.4) | 23.3 (21.7-24.9) | N/A | 1.4 (1-1.9) | Unreliable (0.6-1.8) |
| 2014 | 3.4 (3.1-3.6) | 22.5 (21-24) | Unreliable (2.2-9.1) | 1.8 (1.4-2.3) | N/A |
| 2015 | 3.4 (3.1-3.6) | 23.6 (22-25.1) | N/A | 1.5 (1.1-2) | Unreliable (0.5-1.4) |
| 2016 | 3.3 (3.1-3.5) | 24 (22.4-25.5) | Unreliable (2-7.5) | 1.7 (1.3-2.2) | Unreliable (0.6-1.6) |
| 2017 | 3.5 (3.3-3.7) | 23.6 (22.1-25.1) | N/A | 1.7 (1.3-2.2) | Unreliable (0.6-1.6) |
| 2018 | 3.6 (3.4-3.8) | 22.8 (21.3-24.3) | Unreliable (2.4-8.5) | 1.6 (1.2-2) | 1.3 (0.9-1.9) |
| 2019 | 3.7 (3.4-3.9) | 23 (21.5-24.5) | N/A | 1.8 (1.4-2.3) | 1 (0.6-1.5) |
| 2020 | 4.1 (3.8-4.3) | 28.4 (26.8-30) | Unreliable (2.3-8.1) | 2.1 (1.7-2.6) | 0.9 (0.6-1.4) |
