# Supplementary material for: Shifting temporal trends and disparities in sarcoidosis mortality in the United States: A retrospective analysis from 1999 to 2020
Source: PLoS One. 2025 Jan 10;20(1):e0317237. doi: 10.1371/journal.pone.0317237 (PMC11723600; doi:10.1371/journal.pone.0317237)
Supplement: Attachment — Submitted filename: Review 29.pdf [file pone.0317237.s009.pdf]

Title: Disparities in Mortality from Sarcoidosis by Age, Gender, Ethnoracial Background, and Housing Status: A Retrospective Analysis from 1999 to 2020

Journal: PLOS ONE

Reviewer: Seyed Amirhossein Mazhari

Reviewer's report:

This solid manuscript provides a valuable analysis of sarcoidosis-related mortality trends in the America, covering critical disparities based on multiple variables (age, gender, ethnicity) over a 21 year duration. It is well-constructed and supported by extensive use of CDC data and statistical methodologies.

The manuscript is well-structured and well-analyzed and provides paramount results. The figures are greatly depicted and are structured and clear. The Discussion section is comprehensive and offers a detailed analysis. Furthermore, the study's limitations are effectively addressed.

General comments:

Strengths:

1:
Conclusion part is appropriately detailed and comprehensive. And provides a full overview of the whole article.

2:
Additionally, limitations of the study are provided in a separate and dedicated section, which is highly informative.

Minor revision points:

3:
Clarity of Figures 1-6, while informative, could benefit from more detailed explanations and consistent formatting to improve readability.

4:
It is advised to incorporate an additional section following the conclusion to clearly define and list all abbreviations used in the article.

Thanks to the authors for their hard work on this Study. It’s a pleasure to review such a contribution to the field.
