# Supplementary material for: Shifting temporal trends and disparities in sarcoidosis mortality in the United States: A retrospective analysis from 1999 to 2020
Source: PLoS One. 2025 Jan 10;20(1):e0317237. doi: 10.1371/journal.pone.0317237 (PMC11723600; doi:10.1371/journal.pone.0317237)
Supplement: Attachment — Submitted filename: Review 29a.pdf [file pone.0317237.s011.pdf]

Title: Disparities in Mortality from Sarcoidosis by Age, Gender, Ethnoracial Background, and Housing Status: A Retrospective Analysis from 1999 to 2020

Journal: PLOS ONE

Reviewer: Seyed Amirhossein Mazhari

Reviewer's report:

This solid manuscript provides a valuable analysis of sarcoidosis-related mortality trends in the America, covering critical disparities based on multiple variables (age, gender, ethnicity) over a 21 year duration. It is well-constructed and supported by extensive use of CDC data and statistical methodologies.

The manuscript is well-structured and well-analyzed and provides paramount results. The figures are greatly depicted and are structured and clear. The Discussion section is comprehensive and offers a detailed analysis. Furthermore, the study's limitations are effectively addressed.

Conclusion part is appropriately detailed and comprehensive. And provides a full overview of the whole article. Additionally, limitations of the study are provided in a separate and dedicated section, which is highly informative.

Some Minor revisions points have been suggested and they all have been addressed appropriately.

Minor revision points:

1:
Clarity of Figures 1-6, while informative, could benefit from more detailed explanations and consistent formatting to improve readability.

More informative explanations have been added for Figures.

2:
It is advised to incorporate an additional section following the conclusion to clearly define and list all abbreviations used in the article.

A new section is created and lists all the abbreviated terms throughout the text.

Thanks to the authors for their hard work on this Revision. It’s a pleasure to review such a contribution to the field.
