# Supplementary material for: Shifting temporal trends and disparities in sarcoidosis mortality in the United States: A retrospective analysis from 1999 to 2020
Source: PLoS One. 2025 Jan 10;20(1):e0317237. doi: 10.1371/journal.pone.0317237 (PMC11723600; doi:10.1371/journal.pone.0317237)
Supplement: Attachment — Submitted filename: R2R PONE R1 Sarcoidosis.docx [file pone.0317237.s010.docx]

Dear Respectful Editor and Reviewers,

Thank you for your feedback on our manuscript titled “Shifting Temporal Trends and Disparities in Sarcoidosis Mortality in the United States: A Retrospective Analysis from 1999 to 2020.” We appreciate your recognition of the study's strengths and the valuable insights you provided.

Below we are attaching our in-depth response to each query by the reviewers:

1. ***Editor’s Comments:***

I would like to congratulate the authors for this good paper.

I have some comments to be addressed by the authors:

1-You included 2020 in your study and reflected it on COVID-19 pandemic. What is the last date of the recruitment period in 2020? As WHO declared the pandemic in March 2020.

2-If you have the cause of death, it would be worth noting to report it to exclude if it is COVID-19 related or not, as well as, to know the trends of causes of death. If you can not access it, it is not recommended to report that it is related to COVID-19 directly or indirectly.

-> Dear Editor,

The CDC Wonder database includes data up to the end of each calendar year, with 2020 data extending through the last day of December. However, as per your appropriate suggestion in point 2, the CDC Wonder database does not provide information on causes of death. Accordingly, we have removed any direct or indirect mentions of COVID-19 as a cause, as well as references to it in any hypothesis statements. We would be happy to make any further changes you suggest.

We have added this to our discussion, instead of the COVID-19 hypothesis:

“One possible reason for this recent inflection point could be variations in the coding practices for sarcoidosis-related mortality by physicians, as well as an increased rate of diagnosis and documentation of mortality facilitated by advanced imaging modalities such as high-resolution computed tomography and positron emission tomography scans [19]. Furthermore, despite advancements in therapeutics, adequate management for advanced fibrocystic cases remains unavailable [20], which may further contribute to our findings. Further prospective studies with standard guidelines need to be developed to manage more complex cases of sarcoidosis. Sex disparities were notable, with women generally experiencing higher mortality rates than men, except during the stabilization period, possibly due to hormonal factors [21]. We hypothesize that the sharp increase in mortality among men during 2018-2020 may relate to delayed diagnosis and higher baseline comorbidity rates, and increased vulnerability during the pandemic [22].

To better understand these shifting trends, further research is essential. Cohort studies comparing outcomes between patients with and without COVID-19, along with investigations into healthcare access and treatment continuity during the COVID-19 pandemic, are critical. These efforts will help determine whether the observed mortality increase and the shifting disparities—such as higher mortality in non-metropolitan areas—are partly attributable to the COVID-19 pandemic or other factors. Ultimately, this research will guide targeted interventions to improve outcomes for sarcoidosis patients.”

***2. Review Comments to the Author***

***2.1. Reviewer #1:***

The manuscript is well-structured and well-analyzed and provides paramount results. The figures are greatly depicted and are structured and clear. The Discussion section is comprehensive and offers a detailed analysis. Furthermore, the study's limitations are effectively addressed.

Strengths:

1: Conclusion part is appropriately detailed and comprehensive. And provides a full overview of the whole article.

2: Additionally, limitations of the study are provided in a separate and dedicated section, which is highly informative.

Minor revision points:

3: Clarity of Figures 1-6, while informative, could benefit from more detailed explanations and consistent formatting to improve readability.

-> Dear reviewer, as per your suggestion, we have now added more detail into the figure caption for each figure in the Figure Legend section of our manuscript after the References.

4: It is advised to incorporate an additional section following the conclusion to clearly define and list all abbreviations used in the article.

-> Dear reviewer, a new subheader after the conclusion section for Abbreviations has been created as per your suggestion.

Thanks to the authors for their hard work on this Study. It’s a pleasure to review such a contribution to the field.

-> Dear Reviewer, thank you for your thoughtful comments regarding the strength and novelty of our manuscript.

***2.2. Reviewer #2:***

Overall it's a well written paper and easy to understand. There is not much comment from me except to suggest to put all the figures after the description text. eg. figure 1 should be following the text for sarcoidosis related AAMR stratified by gender.

-> Dear reviewer, thank you for your suggestions. We have added all figures separately as TIFF file in the editorial manager as per the journal’s requirement. However, we have provided the figure caption right after the text, and an additional figure legend at end of the manuscript as per your suggestion.

Thank you,

Mah I Kan Changez, MBBS

Yale University

[Mahikan.changez@yale.edu](mailto:Mahikan.changez@yale.edu)
