# Supplementary material for: Shifting temporal trends and disparities in sarcoidosis mortality in the United States: A retrospective analysis from 1999 to 2020
Source: PLoS One. 2025 Jan 10;20(1):e0317237. doi: 10.1371/journal.pone.0317237 (PMC11723600; doi:10.1371/journal.pone.0317237)
Supplement: Attachment — Submitted filename: R2R PONE R2 Sarcoidosis.docx [file pone.0317237.s012.docx]

Dear Respectful Editor and Reviewers,

Thank you for your feedback on our manuscript titled “Shifting Temporal Trends and Disparities in Sarcoidosis Mortality in the United States: A Retrospective Analysis from 1999 to 2020.” We appreciate your recognition of the study's strengths and the valuable insights you provided.

Below we are attaching our in-depth response to each query by the reviewers:

Reviewer #1: Some Minor revisions points have been suggested and they all have been addressed

appropriately.

Minor revision points:

Thanks to the authors for their hard work on this Revision. It’s a pleasure to review such a

contribution to the field.

-> Dear Reviewer, Thank you for your suggestions and for your feedback that the work is ready for publication.

Reviewer #3: I appreciate your response to the editor’s suggestion regarding the deletion of content related to COVID-19 mortality. However, I noticed that this content is still present in the abstract and keywords. I recommend that you replace it in these sections as you did in the manuscript and remove it from the keywords, as it is not central to the core of the research. Additionally, on page 8, you used the abbreviation “AAPC.” If this was intended to be “APC,” please correct it. If not, kindly provide an explanation for “AAPC” in the abbreviation list. Thank you for your efforts.

-> Dear Reviewer,

We have updated the Abstract and removed the COVID-19 section to reflect the changes made during the Round 1 revisions, which we had inadvertently omitted from the submission portal. Additionally, we have revised the keywords and changed “AAPC” to “APC” in accordance with your comments. Thank you for your valuable feedback.

Please let us know if you’d like any further changes.

Thank you,

Mah I Kan Changez, MBBS

Yale University

[Mahikan.changez@yale.edu](mailto:Mahikan.changez@yale.edu)
